# Patterns of Insulin Secretion During First-Phase Insulin Secretion in Normal Chinese Adults

**DOI:** 10.3389/fendo.2021.738427

**Published:** 2021-11-17

**Authors:** Tao Yuan, Shuoning Song, Tianyi Zhao, Yanbei Duo, Shihan Wang, Junxiang Gao, Shixuan Liu, Yingyue Dong, Rui Li, Yong Fu, Weigang Zhao

**Affiliations:** Department of Endocrinology, Key Laboratory of Endocrinology of Ministry of Health, Peking Union Medical College Hospital, Chinese Academy of Medical Science and Peking Union Medical College, Beijing, China

**Keywords:** normal glucose tolerance, type 2 diabetes mellitus, intravenous glucose tolerance test, first-phase insulin secretion, insulin sensitivity

## Abstract

**Background:**

The increase in diabetes worldwide is alarming. Decreased acute insulin response to intravenous glucose tolerance test (IVGTT) during first-phase insulin secretion (FPIS) is a characteristic of diabetes. However, knowledge of the insulin secretion characteristics identified by different time to glucose peak in subjects with different metabolic state is sparse.

**Aims:**

This study aimed to find different patterns of FPIS in subjects with normal glucose tolerance (NGT) and analyzed the relationship between insulin secretion patterns and the risk for development of type 2 diabetes mellitus (T2DM).

**Methods:**

A total of 126 subjects were divided into three groups during a 10-min IVGTT, including NGT with time to glucose peak after 3 min (G1, n = 21), NGT with time to glucose peak at 3 min (G2, n = 95), and prediabetes or diabetes with time to glucose peak at 3 min (G3, n = 10). Glucose, insulin, and C-peptide concentrations at 0, 3, 5, 7, and 10 min during the IVGTT were tested. IVGTT-based indices were calculated to evaluate the insulin secretion and insulin sensitivity.

**Results:**

Age, body mass index (BMI), waist-to-hip ratio, triglyceride (TG), and hemoglobin A1c (HbA1c) of subjects were gradually higher, while high-density lipoprotein cholesterol (HDL-C) was gradually lower from G1 to G3 (*p* for linear trend <0.05), and the differences between G1 and G2 were also statistically significant (*p* < 0.05). Glucose peak of most participants in G1 converged at 5 min, and the curves shape of insulin and C-peptide in G2 were the sharpest among three groups. There was no significant difference in all IVGTT-based indices between G1 and G2, but AUC_Ins_, AUC_Ins_/AUC_Glu_, and △Ins_3_/△Glu_3_ in G2 were the highest, and the *p-*value for linear trend of those indices among three groups were statistically significant (*p* < 0.05).

**Conclusions:**

Two patterns of FPIS were in subjects with NGT, while subjects with later time to glucose peak during FPIS might be less likely to develop T2DM in the future.

## Introduction

Diabetes is one of the most common metabolic diseases worldwide, the prevalence of which in 2019 is estimated to be 9.3% (463 million people), rising to 10.2% (578 million) by 2030, and half of the people do not know that they have diabetes. Given the present trends, the number of people with diabetes will increase to a staggering 700 million (10.9%) by 2045, being accompanied by a huge economic burden (1). The pathogenesis of type 2 diabetes (T2DM) mellitus is complex, but it is acknowledged that the combination of defective insulin secretion and insulin resistance plays a critical role in the development of T2DM (2). β-cells respond to increased plasma glucose with a biphasic insulin secretion, appearing a sharp first-phase peak and a slowly rising second phase (3). First-phase insulin secretion (FPIS) plays a significant role in maintaining plasma glucose homeostasis, and decline or loss of FPIS is common in T2DM. It seems that the restoration of FPIS and improvement of plasma glucose in T2DM is interacted, which has thus prompted many studies into its regulation (4, 5).

Intravenous glucose tolerance testing (IVGTT) is a common test of β-cell function. Acute increased plasma glucose can induce an apparent FPIS, and insulin and C-peptide responses are also monitored. Hulman et al. (6) analyzed data from participants without diabetes from the observational cohort from the European Group for the Study of Insulin Resistance and identified four different glucose response patterns according to time to glucose peak and glucose response curve during oral glucose tolerance test (OGTT). However, studies related to FPIS identified by time to glucose peak during IVGTT are sparse. In this study, we examined the insulin secretion in subjects with different metabolic state and glucose response curve during FPIS through IVGTT, aiming to find different patterns of FPIS in subjects with normal glucose tolerance (NGT) and analyzed the relationship between insulin secretion patterns and the risk for development of T2DM.

## Materials and Methods

### Study Population

A total of 126 participants, including 116 healthy subjects, 5 patients with prediabetes, and 5 patients with T2DM, were consecutively recruited in this study in the Department of Endocrinology of Peking Union Medical College Hospital from November 2020 to April 2021. The exclusion criteria were as follows: (1) acute complications of diabetes, (2) hepatic or renal disease and systemic corticosteroid treatment, and (3) women who were currently pregnant and breastfeeding. All experimental protocols were approved by the Ethics Committee of Peking Union Medical College Hospital and performed in accordance with the Declaration of Helsinki as revised in 2013. Written informed consent was obtained from each participant.

### Measurements

Clinical parameters such as height, body weight, waist circumferences (Wc), hip circumferences (Hc), biceps circumferences (Bc), thigh circumferences (Tc), systolic blood pressure (SBP), and diastolic blood pressure (DBP) were collected. Systolic and diastolic blood pressure was measured three times by a standard mercury manometer with the subjects seated. The past and family history were recorded using a questionnaire.

Overnight fasting blood samples were collected before IVGTT. Alanine transaminase (ALT), aspartate aminotransferase (AST), serum creatinine (SCr), uric acid (UA), hemoglobin A1c (HbA1c), triglyceride (TG), total cholesterol (TC), high-density lipoprotein cholesterol (HDL-C), and low-density lipoprotein cholesterol (LDL-C) were measured in all subjects.

After an overnight fasting, IVGTT was performed in all subjects. Glucose solution (25 g; 50 ml of 50% glucose) was injected intravenously within 1 min, and blood samples were obtained from the opposite arm before (0 min) and at 3, 5, 7, and 10 min after glucose infusion for plasma glucose, insulin, and C-peptide determinations.

Seven patients among subjects with prediabetes or diabetes underwent hyperinsulinemic–euglycemic glucose clamp test before IVGTT. There was more than 1-h interval between the two tests. However, plasma glucose at 0 min before IVGTT was lower than their fasting plasma glucose (FPG). To revise the plasma glucose level value without changing the glucose curve shape, FPG was recorded as plasma glucose at 0 min (Glu0), and plasma glucose at each point was recorded as real plasma glucose + (FPG-Glu0).

Related calculation formula were as follows. Body mass index (BMI) was determined by dividing body weight in kilograms by height in meters squared. Waist-to-hip ratio (WHR) was defined as Wc/Hc, and biceps-to-thigh ratio (BTR) was defined as Bc/Tc. The area under curve (AUC) of glucose (AUC_Glu_), insulin (AUC_Ins_), and C-peptide (AUC_C-p_) were calculated by GraphPad Prism (Version 8.0). The following IVGTT-based indices of insulin secretion were studied (7): (a) insulinogenic index was calculated as (Ins_y_ − Ins_0_)/(Glu_y_ − Glu_0_), where Ins_y_ and Glu_y_ represent insulin and glucose values, respectively, at time y min during the IVGTT, and (b) the ratio of the total AUC_Ins_ to the total AUC_Glu_ was calculated to evaluate insulin sensitivity.

### Statistical Analysis

All analyses were conducted using the statistical program SPSS (version 24, SPSS, Chicago, IL). Continuous variables were tested for normality of distribution. Variables with approximately normal distributions are presented as mean ± SD, and those with skewed distributions are presented as median (interquartile range). Categorical variables are presented as percentage (number). One-way ANOVA with least significant difference (LSD) *post-hoc* test was performed for multiple comparisons. To determine whether there was a significant graded change of variables among the three groups, the *p*-value for the linear trend was calculated. Relationships between variables were assessed using Spearman’s correlation analysis. Receiver operating characteristic (ROC) curve analysis was used to determine the optimal cutoff points of age and BMI for the prediction of glucose peak time at 3 min. Statistical significance was inferred from two-sided *p*-values <0.05.

## Results

One hundred twenty-six subjects were divided into three groups according to different glucose metabolic state and time to glucose peak, including 21 subjects with NGT and time to glucose peak after 3 min (G1), 95 subjects with NGT and time to glucose peak at 3 min (G2), and 10 subjects with prediabetes or diabetes mellitus and time to glucose peak at 3 min (G3). A comparison of clinical and laboratory characteristics among the three groups is shown in **Table 1**. Age, BMI, WHR, TG, and HbA1c of subjects in G1 and G2 were higher than those in G3 (all *p* < 0.05) and were gradually higher from G1 to G3 (*p* for linear trend < 0.05). HDL-C in G1 was lower than G2 and G3 (*p* < 0.05), and the decreasing trend was statistically different (*p* for linear trend <0.05). UA level among the three groups was increasing, although the difference was not significant (*p* > 0.05).

**Table 1:** Clinical and laboratory characteristics of participants by different glucose metabolic state and time to glucose peak at baseline.

Variables	G1 (n = 21)	G2 (n = 95)	G3 (n = 10)	*p* for linear trend
Age	26 (25,33)^a^	32 (25,39)^b^	44 (37,47)^c^	**<0.001**
Male	28.6% (6)	41.1% (39)	50% (5)	0.449
BMI (kg/m^2^)	21.28 (19.79,23.05)^a^	23.03 (21.22,25.00)^b^	25.20 (23.31,28.70)^c^	**0.001**
WHR	0.80 (0.75,0.84)	0.82 (0.77,0.87)^b^	0.92 (0.84,1.01)^c^	**<0.001**
BTR	0.52 (0.49,0.57)^a^	0.57 (0.52,0.61)	0.58 (0.56,0.59)	0.090
SBP (mmHg)	112.67 (103.33,121.33)	115 (106,123)	120 (109,132)	0.218
DBP (mmHg)	73.00 (64.67,76.33)	72 (66,83)	75 (70,85)	0.121
ALT (U/L)	10 (9,20)	17 (13,25)	26 (16,31)	0.179
AST (U/L)	16 (13,19)	19 (15,24)	26 (21,33)	0.703
SCr (umol/L)	68 (59,75)	66 (59,80)	69 (56,78)	0.993
UA (umol/L)	279 (269,352)	307 (247,381)	337 (302,373)	0.242
TC (mmol/L)	4.77 ± 0.65	4.55 ± 0.91^b^	5.36 ± 0.69	0.082
TG (mmol/L)	0.86 (0.52,1.29)	0.86 (0.59,1.46)^b^	1.63 (1.08,2.96)^c^	**<0.001**
HDL-C (mmol/L)	1.44 ± 0.40^a^	1.28 ± 0.30	1.12 ± 0.25^c^	**0.009**
LDL-C (mmol/L)	2.64 ± 0.50	2.71 ± 0.78	3.18 ± 0.64	0.068
HbA1c (%)	5.10 (5.00,5.20)	5.10 (5.00,5.40)^b^	6.05 (5.50,6.60)^c^	**<0.001**

Data are presented as mean ± SD or as median (interquartile range) or percentage (number).

BMI, body mass index; WHR, waist-to-hip ratio; BTR, biceps-to-thigh ratio; SBP, systolic blood pressure; DBP, diastolic blood pressure; ALT, alanine transaminase; AST, aspartate aminotransferase; SCr, serum creatinine; UA, uric acid; TC, total cholesterol; TG, triglyceride; HDL-C, high-density lipoprotein cholesterol; LDL-C, low-density lipoprotein cholesterol; HbA1c, hemoglobin A1c.

a Difference between group 1 and group 2 was statistically significant (p_1–2_ < 0.05).

b Difference between group 2 and group 3 was statistically significant (p_2–3_ < 0.05).

c Difference between group 1 and group 3 was statistically significant (p_1–3_ < 0.05). P values < 0.05 were shown in bold.

**Figure 1:**
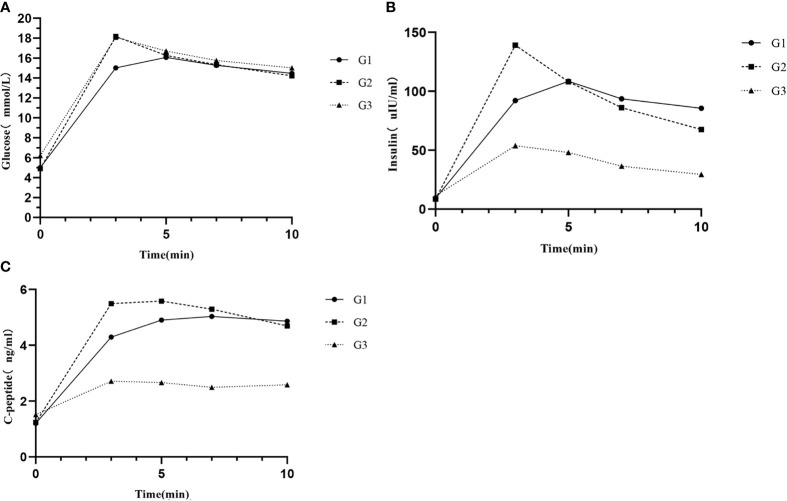
**(A)** Glucose responses, **(B)** insulin responses, and **(C)** C-peptide responses during IVGTT among different groups. G1, subjects with normal glucose tolerance (NGT) and time to glucose peak after 3 min; G2, subjects with NGT and time to glucose peak at 3 min; and G3, subjects with prediabetes or diabetes mellitus and time to glucose peak at 3 min.

The glucose curves among the three groups are plotted in **Figure 1A** alongside their corresponding insulin and C-peptide trajectories (**Figures 1B, C**). Glucose peak at 3 min in G2 was the highest with a sharp decreased trend following, which consisted with the trends of insulin and C-peptide. The glucose peak in G3 also appeared at 3 min, whereas the glucose peak of most participants in G1 was at 5 min. Insulin peak time among the three groups were roughly consistent with the glucose peak time. C-peptide peak time was at 7 min in G1 an G2, while it was at 3 min in G3. On the contrary to G2, insulin and C-peptide curves in G1 and G3 were relatively flat after glucose peak. Insulin and C-peptide were significantly lower in G3 among the three groups. There were no significant differences in glucose, insulin and C-peptide at other points in time between G1 and G2 except at 3-min point (**Supplemental Table S1**).

**Table 2** shows different IVGTT-based indices during 10-min IVGTT. There were no significant differences in all indices between G1 and G2. AUC_Ins_, AUC_Ins_/AUC_Glu_, △Ins_3_/△Glu_3_, and △Ins_10_/△Glu_10_ in G1 and G2 were all significantly higher than those in G3 (*p* < 0.05), and △Ins_10_/△Glu_10_ was gradually decreased from G1 to G3 (*p* for linear trend < 0.05). AUC_Ins_, AUC_Ins_/AUC_Glu_, and△Ins_3_/△Glu_3_ in G2 were the highest, and the *p* value for the linear trend of those indices among the three groups was statistically significant.

**Table 2:** Comparison of IVGTT-based indices among three groups.

Variables	G1 (n = 21)	G2 (n = 95)	G3 (n = 10)	*p* for linear trend
AUC_Glu_	137.07 ± 24.97	144.79 ± 20.87	151.44 ± 36.16	0.107
AUC_Ins_	787.40 (581.80,1077.00)	891.70 (592.90,1345.00)^b^	389.00 (221.10,562.10)^c^	**0.038**
AUC_C-p_	43.61 (34.27,51.14)	47.15 (36.51,66.38)^b^	23.21 (20.96,43.92)	0.096
AUC_Ins_/AUC_Glu_	5.73 (4.30,7.58)	6.00 (3.76,10.49)^b^	2.65 (1.20,4.18)^c^	**0.023**
△Ins_3_/△Glu_3_	8.00 (6.48,13.93)	9.21 (6.17,14.13)^b^	3.44 (1.35, 5.71)^c^	**0.002**
△Ins_10_/△Glu_10_	6.76 (5.08,9.51)	6.23 (3.70,10.65)^b^	1.85 (1.34,5.88)^c^	**0.025**

Data are presented as mean ± SD or as median (interquartile range).

AUC, area under curve; Glu, plasma glucose; Ins, insulin; C-p, C-peptide; △Ins_y_/△Glu_y_ = (Ins_y_ − Ins_0_)/(Glu_y_ − Glu_0_), Ins_y_ and Glu_y_ represented insulin and glucose values, respectively, at time y min during the intravenous glucose tolerance test.

a Difference between group 1 and group 2 was statistically significant(p_1–2_ < 0.05).

b Difference between group 2 and group 3 was statistically significant (p_2–3_ < 0.05).

c Difference between group 1 and group 3 was statistically significant (p_1–3_ < 0.05). P values < 0.05 were shown in bold.

In consideration of the large sample difference of participants between G1 and G2, and the different glucose metabolic states between prediabetes and T2DM, a random selection of 21 subjects from G2 as G2r were compared to G1, and participants in G3 were divided into G3a (prediabetes, n = 5) and G3b (T2DM, n = 5); hence a more detailed grouping is shown in **Supplemental Table S2**. The trends of variables among the four groups were similar to these in the initial groups and the statistical significance still holds.

A further analysis on relationships between age, BMI, and glucose metabolic characteristics is shown in **Supplemental Table S3**, and receiver operating characteristic (ROC) curves (**Supplemental Figure S1**) were used to trying to determine the optimal cutoff points of age and BMI for the prediction of different patterns of insulin secretion. The cutoff points of age, BMI, and age combined with BMI for predicting glucose peak at 3 min were 26.5, 21.35, and 0.872 (AUC, 0.689; 95% CI, 0.579–0.799, *p* = 0.006; AUC, 0.695; 95% CI, 0.576-0.813, *p* = 0.005; AUC, 0.742; 95% CI 0.642–0.842, *p* < 0.001), respectively.

## Discussion

In this study, we not only demonstrated a new way of evaluating FPIS by glucose responses during IVGTT but also provided the metabolic state of subjects with different time to glucose peak and insulin curve shapes. According to our results, glucose peak at 3 min and after 3 min might be two patterns of FPIS in adults with NGT, but the former one might be a T2DM susceptibility pattern while subjects with later glucose peak might be less likely to develop metabolic disorders in future. To our knowledge, we were the first study to propose this view in subjects with NGT.

Insulin is released from the pancreatic β cells to respond to increased plasma glucose concentration with a biphasic pattern. Although the mechanisms of biphasic pattern of insulin remain incompletely understood, insulin granules with differing release probabilities is an acceptable interpretation. The released competent insulin granules are adjacent to the plasma membranes, releasing to peripheral blood immediately to respond to elevated plasma glucose, contributing to a sharp increase in plasma insulin, which is known as FPIS (8). The FPIS consists of a brief spike lasting about 10 min followed by a second phase, which is slow but can reach a plateau at 2–3 h (9).

Previous studies have reported that decreased FPIS is an early marker of β-cell dysfunction during development of T2DM, and even mild chronic hyperglycemia is also harmful for β-cell function (10, 11). The classic interpretation of the decreased insulin secretion is the lack of releasable insulin granules in the critical pool (12). A recent study reported that human insulin secretion and exocytosis critically depend on the availability of membrane-docked granules and that T2DM is associated with a strong reduction in granule docking. Besides, the recovery of releasable granules is much slower in cells from subjects with T2DM than in those of subjects with NGT (13). Hong et al. (14) evaluated FPIS in Chinese obese subjects with different categories of impaired glucose regulation and found the FPIS in the NGT group was significantly greater than that in healthy lean control group, but then, it was progressively decreased in impaired glucose tolerance and T2DM group. In the present study, insulin and C-peptide level in G3, especially in G3b (participants with T2DM), were much lower than those in subjects with NGT, implying an impaired β-cell function, which was consistent with previous studies.

However, few studies reported the potential difference among healthy adults identified by different glucose responses patterns. In the present study, although the comparison of glucose, insulin, and C-peptide during FPIS did not have significant difference between G1 and G2 except those at 3-min point, subjects in G1 appeared to have more flat curve no matter the glucose, insulin, or C-peptide than in G2, and the *p* for linear trend of AUC_Ins_, AUC_Ins_/ACU_Glu_, and △Ins_10_/△Glu_10_ among the three groups were statistically significant, which implied that there might be two patterns of FPIS in subjects with NGT. Relatively higher AUC_Ins_ and AUC_Ins_/ACU_Glu_ suggested higher insulin response and lower insulin sensitivity, respectively, which were similar to FPIS in African Americans (AAs) compared with non-Hispanic whites (NHWs) (15, 16). Previous studies found that healthy AAs have a higher insulin response following an intravenous glucose load when compared with NHWs (17). A traditional view is that lower insulin sensitivity may be a driver of compensatory increases in β-cell function and hyperinsulinemia (18). The other opinion is that increased insulin secretion is primary hypersecretion independent of compensatory response to insulin resistance (19). Recently, Armiyaw et al. (20) did further studies among AAs and NHWs by IVGTT and hyperinsulinemic–euglycemic glucose clamp technique to support this opinion. According to our study, it seems that the two patterns of first-phase insulin and C-peptide response exist not only in different races but also in healthy participants in the same race.

The curves of subjects in G2 suggested faster and higher insulin secretion and C-peptide compared with subjects in G1, and relatively lower △Ins_10_/△Glu_10_ implied the lack of sustainability of the insulin secretion in G2. Thus, the stabilization points of insulin secretion in G1 and G2 might be different. Keiichi Kodama et al. (21) reported that there were ethnic differences in the optimal states in the relationship between insulin sensitivity and insulin response. Even a small change may cause a rapid increase in the amount of insulin secretion required to maintain NGT in Africans. They assumed that this unstable feedback loop may be implicated in this group’s vulnerability of canalization of blood glucose levels. This genetic background of Africans makes them more and differentially susceptible to diabetes than Caucasians. We hypothesized that this difference also exists in the same race. The insulin response in G1 was in the most stable optimum zone during FPIS, while it was located around an unstable extreme point in G2. The other potential interpretation of the difference between G1 and G2 may be related to the insulin granule dynamics in pancreatic β cells (22). Although β-cell function in G2 was still enough to respond to glucose stimulation, recovery of new releasable granule β cells was much slower, and they did not have enough ability to sustain a higher insulin secretion. However, the exact mechanism is not clear and needs further investigation.

There were other results that should be noticed. Hong et al. (23) have reported that Chinese obese subjects with NGT have increased FPIS compared with lean subjects. In this study, we found time to glucose peak in subjects with prediabetes or diabetes at 3 min, and there were also significant differences in some clinical and laboratory characteristics related to metabolic state between NGT subjects with time to glucose peak at and after 3 min. Older age and BMI and lower LDL-C, which implied higher risk of metabolic disorders, were found in G2. It is interesting that compared with NHWs, healthy AAs also have a higher risk for the development of T2DM (24). Given the characteristics of insulin sensitivity, acute insulin response, and insulin secretion curves in G1 and G2 were all similar to those in NHWs and AAs, we provided a hypothesis that time to glucose peak during FPIS may be related to the risk of T2DM. People with later time to glucose peak in G1 might be less likely to develop T2DM in the future. However, follow-up in these subjects is needed to confirm this view.

This study has a few limitations. First, although all our subjects in G1 and G2 were non-diabetic, glucose tolerance state following an oral glucose tolerance test was not assessed directly. Second, several subjects in G3 underwent hyperinsulinemic–euglycemic glucose clamp test before IVGTT. Although there was an interval between the two tests and adjusted glucose levels were used in analysis, it was difficult to totally eliminate potential impact. Moreover, the time points for evaluating glucose and insulin level were not earlier and closer enough. According to a previous study, glucose peak in normal people is usually within 3–6 min in FPIS. In this study, we cannot exclude that glucose peak time in some participants were out of this range and might appear before 3 min, and the actual peak might be missed. Lastly, the relatively small sample size in each group, especially in prediabetes and T2DM groups, was an additional limitation. Our finding should be investigated in further large studies.

## Conclusions

In conclusion, different time to glucose peak implied two patterns of insulin secretion during FPIS in subjects with NGT, which might be related to varying degrees of risk for T2DM. People with later time to glucose peak might be less likely to develop T2DM in the future.

## Data Availability Statement

The raw data supporting the conclusions of this article will be made available by the authors, without undue reservation.

## Ethics Statement

The studies involving human participants were reviewed and approved by The Ethics Committees of Peking Union Medical College Hospital. The patients/participants provided their written informed consent to participate in this study.

## Author Contributions

Conceptualization: TY, SS, and WZ. Investigation: TY, SS, TZ, YaD, SW, JG, SL, YiD, RL, and YF. Methodology: TY and SS. Clinical data collection: SS, TZ, YaD, SW, JG, SL, YiD, RL, and YF. Writing—original draft: TY and SS. Writing—review editing: WZ and TY. Supervision: WZ and TY. All authors contributed to the article and approved the submitted version.

## Funding

This study was supported by CAMS Innovation Fund for Medical Science (CIFMS), the Non-profit Central Research Institute Fund of Chinese Academy of Medical Sciences under Grant No. 2016-I2M-4-001 (to TY).

## Conflict of Interest

The authors declare that the research was conducted in the absence of any commercial or financial relationships that could be construed as a potential conflict of interest.

## Publisher’s Note

All claims expressed in this article are solely those of the authors and do not necessarily represent those of their affiliated organizations, or those of the publisher, the editors and the reviewers. Any product that may be evaluated in this article, or claim that may be made by its manufacturer, is not guaranteed or endorsed by the publisher.
