# Supplementary material for: Patterns of Insulin Secretion During First-Phase Insulin Secretion in Normal Chinese Adults
Source: Front Endocrinol (Lausanne). 2021 Nov 17;12:738427. doi: 10.3389/fendo.2021.738427 (PMC8635794; doi:10.3389/fendo.2021.738427)
Supplement: Supplementary file 1 [file DataSheet_1.docx]

**Supplemental Tables**

**Supplemental Table 1. Glucose, insulin and C-peptide during first-phase insulin secretion among three groups**

| Variables | G1(n=21) | G2 (n=95) | G3（n=10） | *P* for linear trend |
| --- | --- | --- | --- | --- |
| Glu0(mmol/L) | 5.00(4.87,5.20) | 4.90(4.50,5.20)^b^ | 6.20(5.60,6.90)^c^ | **＜0.001** |
| Glu3(mmol/L) | 15.01±3.16^a^ | 18.17±2.80 | 18.09±4.22^c^ | **0.008** |
| Glu5(mmol/L) | 16.07±3.34 | 16.26±2,43 | 16.71±4.04 | 0.547 |
| Glu7(mmol/L) | 15.27±2.88 | 15.33±2.33 | 15.76±3.83 | 0.618 |
| Glu10(mmol/L) | 14.47±2.50 | 14.22±2.21 | 15.02±3.93 | 0.553 |
| Ins0(uIU/ml) | 9.20(7.00,11.90) | 8.60(5.90,12.10) | 11.34(8.10,16.60) | 0.126 |
| Ins3(uIU/ml) | 92.10(76.30,134.10)^a^ | 139.00(85.83,197.00)^b^ | 53.80(27.50,77.94) | 0.079 |
| Ins5(uIU/ml) | 108.40(70.80,150.10) | 108.10(70.70,172.00)^b^ | 48.00(21.40,69.60)^c^ | **0.030** |
| Ins7(uIU/ml) | 93.60(56.30,123.20) | 86.10(57.95,139.20)^b^ | 36.40(22.80,54.40)^c^ | **0.024** |
| Ins10(uIU/ml) | 85.50(45.30,100.20) | 67.50(46.00,113.90)^b^ | 29.40(26.10,49.16)^c^ | **0.043** |
| C-p0(ng/ml) | 1.20(1.09,1.36) | 1.23(0.91,1.71)^b^ | 1.51(1.30,1.89)^c^ | **0.015** |
| C-p3(ng/ml) | 4.29(3.54,4.35)^a^ | 5.49(4.18,8.25)^b^ | 2.71(2.10,5.10) | 0.325 |
| C-p5(ng/ml) | 4.90(3.78,6.44) | 5.58(3.80,7.65)^b^ | 2.66(2.07,4.61)^c^ | **0.047** |
| C-p7(ng/ml) | 4.92(3.78,5.57) | 5.29(3.37,6.89)^b^ | 2.49(2.24,2.53)^c^ | **0.022** |
| C-p10(ng/ml) | 4.98(3.59,5.96) | 4.69(3.63,6.47)^b^ | 2.58(2.12,4.34) | **0.039** |

Data are presented as mean±SD or median (interquartile range)

Glu_y_, Ins_y_ and C-p_y_ represent plasma glucose, insulin and C-peptide values, respectively, at time y min during intravenous glucose tolerance test.

^a^: difference between group 1 and group 2 was statistically significant(*P*_1-2_＜0.05)

^b^: difference between group 2 and group 3 was statistically significant (*P*_2-3_＜0.05)

^c^: difference between group 1 and group 3 was statistically significant (*P*_1-3_＜0.05)**Supplemental Table 2. Clinical and laboratory characteristics as well as IVGTT-based indices of participants by different glucose metabolic state and time to glucose peak at baseline**

| Variables | G1(n=21) | G2r(n=21) | G3a(n=5) | G3b(n=5) | *P* for linear trend |
| --- | --- | --- | --- | --- | --- |
| Age | 26(25,33) | 27(24,36) | 45(42,47) | 42(37,45) | ＜0.001 |
| Male | 28.6%(6) | 38.1%(8) | 40%(2) | 60%(3) | 0.200 |
| BMI(kg/m^2^) | 21.28(19.79,23.05) | 22.53(21.38,27.22) | 26.57(24.00,28.70) | 23.60(23.31,26.40) | 0.004 |
| WHR | 0.80(0.75,0.84) | 0.82(0.78,0.85) | 0.88(0.84,0.95) | 0.96(0.85,1.01) | ＜0.001 |
| BTR | 0.52(0.49,0.57) | 0.54(0.51,0.59) | 0.58(0.56,0.59) | 0.57(0.56,0.59) | 0.129 |
| SBP(mmHg) | 113(103,121) | 120(108,130) | 119(109,125) | 121(114,132) | 0.401 |
| DBP(mmHg) | 73(65,76) | 71(66,81) | 74(74,77) | 76(69,85) | 0.154 |
| ALT(U/L) | 10(9,20) | 17(13,22) | 31(21,53) | 24(13,27) | 0.052 |
| AST(U/L) | 16(13,19) | 16(14,20) | 32(26,33) | 21(20,23) | ＜0.001 |
| SCr(umol/L) | 68(59,75) | 71(60,78) | 77(58,77) | 60(56,78) | 0.854 |
| UA(umol/L) | 279(269,352) | 326(272,386) | 361(355,383) | 305(302,318) | 0.694 |
| TC(mmol/L) | 4.77±0.65 | 4.45±1.04 | 5.50±0.83 | 5.22±0.59 | 0.096 |
| TG(mmol/L) | 0.86(0.52,1.29) | 0.80(0.66,1.14) | 1.84(1.41,2.66) | 1.22(0.93,5.09) | ＜0.001 |
| HDL-C(mmol/L) | 1.44±0.40 | 1.33±0.31 | 1.15±0.22 | 1.09±0.29 | 0.026 |
| LDL-C(mmol/L) | 2.64±0.50 | 2.69±0.73 | 3.48±0.67 | 2.89±0.51 | 0.171 |
| HbA1c(%) | 5.10(5.00,5.20) | 5.10(5.00,5.30) | 5.50(5.30,5.50) | 6.60(6.30,8.50) | ＜0.001 |
| AUC_Glu_ | 137.07±24.97 | 146.14±17.09 | 140.02±24.06 | 162.86±45.14 | 0.071 |
| AUC_Ins_ | 787.40(581.80,1077.00) | 838.80(628.00,1573.00) | 562.10(516.20,618.80) | 172.45(93.81,235.20) | 0.002 |
| AUC_C-p_ | 43.61(34.27,51.14) | 48.78(36.60,64.55) | 43.92(25.53,45.27) | 19.97(14.51,21.96) | 0.003 |
| AUC_Ins_/AUC_Glu_ | 5.73(4.30,7.58) | 5.97(4.08,10.62) | 4.18(3.91,5.29) | 0.96(0.66,1.37) | 0.004 |

Data are presented as mean±SD or as median (interquartile range) or percentage (number)

Abbreviations: BMI, body mass index; WHR, waist-to-hip ratio; BTR, biceps-to-thigh ratio; SBP, systolic blood pressure; DBP, diastolic blood pressure; ALT, alanine transaminase; AST, aspartate aminotransferase; SCr, serum creatinine; UA, uric acid; TG, triglyceride; TC, total cholesterol; HDL-C, high-density lipoprotein cholesterol; LDL-C, low-density lipoprotein cholesterol; HbA1c, hemoglobin A1c; AUC, area under curve; Glu, plasma glucose; Ins, insulin; C-p, C-peptide; △Ins_y_/△Glu_y_= (Ins_y_− Ins_0_)/(Glu_y_ − Glu_0_), Ins_y_ and Glu_y_ represented insulin and glucose values, respectively, at time y min during the intravenous glucose tolerance test.

**Supplemental Table 3. Spearman's correlation of age, BMI and FPIS in participants with normal glucose tolerance**

| Variables | Age | BMI | AUC_Glu_ | AUC_Ins_ | AUC_C-p_ | AUC_Ins_/AUC_Glu_ | |
| --- | --- | --- | --- | --- | --- | --- | --- |
| Age | 1 |  |  |  |  | |  |
| BMI | 0.238* | 1 |  |  |  | |  |
| AUC_Glu_ | 0.054 | -0.419** | 1 |  |  | |  |
| AUC_Ins_ | -0.306** | 0.191* | 0. 870 | 1 |  | |  |
| AUC_C-p_ | -0.277** | 0.299** | 0.120 | 0.905** | 1 | |  |
| AUC_Ins_/AUC_Glu_ | -0.313** | 0.299** | -0.169 | 0.956** | 0.887** | | 1 |

* P＜0.05

**P＜0.001

Abbreviations: BMI, body mass index; AUC, area under curve; Glu, plasma glucose; Ins, insulin; C-p, C-peptide

**Supplemental Figure**


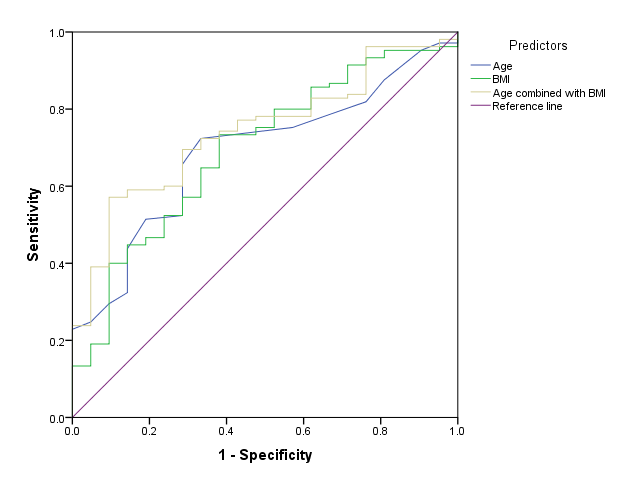


**Supplemental Figure 1. Age and BMI as predictors of glucose peaks at 3 min in all participants**

The cut-off points of age, BMI and age combined with BMI were 26.5, 21.35 and 0.872 (area under the curve[AUC] 0.689, 95% CI 0.579-0.799, P= 0.006; AUC 0.695, 95% CI 0.576-0.813, P= 0.005; AUC 0.742, 95% CI 0.642-0.842, P< 0.001), respectively.
